# Simultaneously Enhancing Efficiency and Stability of Ternary Organic Solar Cells via a Benzothiadiazole-Thieno[3,2-c]isochromene-Based Small-Molecule Donor in the PTB7-Th:PC_71_BM System

**DOI:** 10.3390/molecules31142552

**Published:** 2026-07-22

**Authors:** Wei Tang, Wenjie Zeng, Junjie Liu, Junhui Zhou, Xiaobing Lan

**Affiliations:** Hunan Provincial Key Laboratory of Xiangnan Rare-Precious Metals Compounds Research and Application, School of Chemistry and Environmental Science, Xiangnan University, Chenzhou 423000, China; weitang031@xnu.edu.cn (W.T.); m19312322508@163.com (W.Z.); 17267637801@163.com (J.L.); 17375657187@163.com (J.Z.)

**Keywords:** organic solar cells, small-molecule donor, thieno[3,2-c]isochromene, ternary strategy, device stability

## Abstract

The ternary strategy has emerged as an effective approach to enhance the photovoltaic performance of organic solar cells (OSCs), yet simultaneously improving both efficiency and stability remains a formidable challenge. Herein, we report a small-molecule donor (TiC12), featuring a thieno[3,2-c]isochromene unit, as a third component to fabricate ternary fullerene-based OSCs using the low-cost PTB7-Th:PC_71_BM host matrix. The influence of TiC12 on the active-layer morphology, device efficiency, and long-term stability is systematically investigated. The results reveal that the incorporation of TiC12 enables precise regulation of the blend film morphology, leading to a marked improvement in photovoltaic performance. Consequently, the optimized ternary devices achieve a notable power conversion efficiency (PCE) of 10.42%, which significantly surpasses that of the corresponding binary PTB7-Th:PC_71_BM counterparts (9.16%). More importantly, the ternary system simultaneously exhibits substantially enhanced operational stability, retaining 82.7% of its initial PCE after 2304 h under ambient storage in a N_2_-filled glovebox. This work demonstrates that TiC12, with its unique thieno[3,2-c]isochromene framework, represents a promising third-component candidate for achieving both high efficiency and superior stability in ternary fullerene OSCs.

## 1. Introduction

The escalating global energy demand has propelled organic solar cells (OSCs) into the spotlight as a compelling next-generation photovoltaic technology [1,2,3]. OSCs offer distinct advantages, including low-cost solution processability, lightweight nature, mechanical flexibility, and the potential for large-scale roll-to-roll manufacturing. However, despite these merits, the power conversion efficiency (PCE) and long-term operational stability of OSCs remain critical bottlenecks hindering their commercial viability [4,5,6]. To address these challenges, a variety of strategies have been explored, such as novel acceptor molecular design, interfacial engineering, and innovative device architectures [7,8,9]. Among these approaches, the construction of ternary active layers has emerged as a particularly promising and straightforward tactic to simultaneously boost PCE and stability by integrating a complementary component into a binary host system [10,11,12,13].

Concurrently, the imperative to mitigate environmental issues has intensified research into renewable energy technologies, with OSCs consistently attracting significant interest due to their outstanding performance potential [14]. Remarkably, recent breakthroughs have pushed the PCE of single-junction OSCs beyond the 21% milestone, driven by innovations in acceptor materials, interfacial modification, morphological optimization, and processing techniques [15]. Among these optimization routes, the fine-tuning of bulk-heterojunction (BHJ) morphology is widely regarded as the most complex yet effective lever for governing device performance [16,17,18]. The active-layer nanomorphology is governed by a multitude of interrelated factors, including the solubility, crystallinity, and miscibility of donor/acceptor materials, the choice of processing techniques (e.g., spin-coating, blade-coating, inkjet printing), and the device configuration (e.g., conventional, inverted, or multi-interlayer structures) [19,20]. In practice, only a limited number of blend systems spontaneously yield an optimal morphology, and relying solely on the intrinsic properties of acceptor materials often proves insufficient to achieve the desired nanoscale phase separation and device characteristics. Therefore, the rational design of donor materials, particularly through the ternary strategy, has become a crucial avenue for optimizing photovoltaic performance [21,22].

In this context, the ternary blend approach offers a dual advantage: it not only extends photon harvesting via the complementary absorption spectrum of the third component, but also provides an additional handle for modulating the microstructural evolution of the active layer [23,24,25]. This strategy has been demonstrated as a highly efficient and feasible route to further elevate PCE in OSCs [26,27]. Nevertheless, while high PCE values are now routinely achieved, the intrinsic instability of OSCs remains a formidable obstacle to practical application [28,29]. Addressing this issue by enhancing device durability without sacrificing efficiency, and ideally reducing overall costs, has become a central research focus. Although recent studies have reported stable devices with minimized efficiency losses through tailored material design and interfacial engineering [30,31,32], systematic investigations correlating ternary-component molecular structure with both efficiency and stability are still relatively scarce and warrant further exploration.

**Figure 1:**
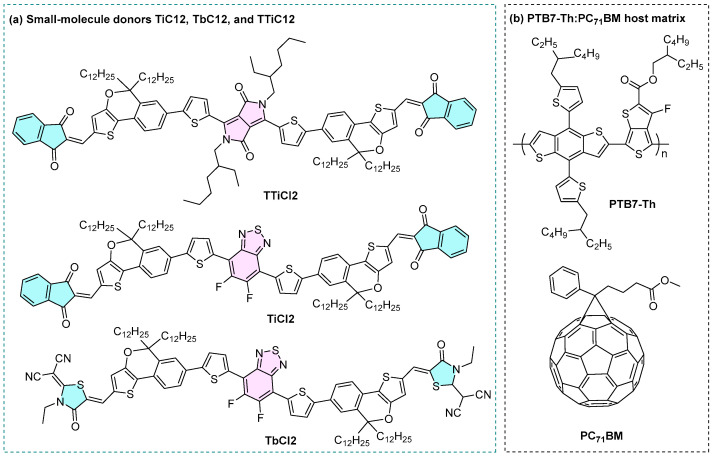
Molecular structure formula of the organic materials (**a**) TTiC12, TiC12, TbC12, (**b**) PTB7-Th and PC_71_BM.

In this work, we systematically investigate the interplay between active-layer morphology, photovoltaic performance, and stability by introducing three structurally related small-molecule donors, designated as TTiC12, TiC12, and TbC12 (Figure 1a), which are based on a thieno[3,2-c]isochromene core functionalized with bis(dodecyl)thiophene arms, into the well-established PTB7-Th:PC_71_BM host matrix (Figure 1b). We particularly focus on elucidating how variations in the molecular core and terminal cell structures of these small-molecule donors influence the nanoscale morphology of the photoactive layer and, consequently, the overall device performance of ternary fullerene OSCs. By leveraging the distinct intermolecular interactions and chemical properties among these components, we demonstrate that the active-layer morphology can be effectively manipulated, thereby providing a viable pathway to simultaneously enhance the efficiency and stability of OSCs. Although this study primarily focuses on fullerene derivatives, these materials are theoretically also highly promising for non-fullerene acceptor systems. Notably, TiC12 and TbC12 exhibit high molar extinction coefficients and readily tunable energy levels, which make them attractive third-component candidates for cutting-edge non-fullerene organic solar cells beyond fullerene-based host systems. We plan to conduct follow-up studies in the future to further verify their general photovoltaic applicability.

## 2. Results and Discussion

### 2.1. Photophysical Property

**Figure 2:**
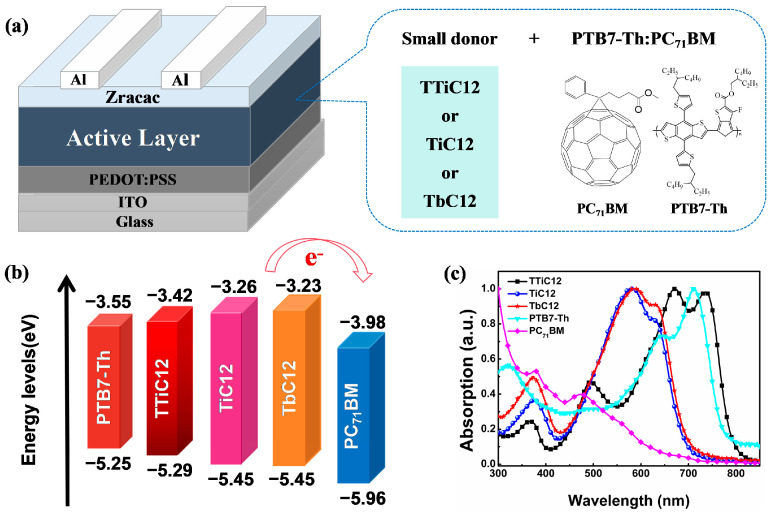
(**a**) Device structure diagram, (**b**) energy-level diagram, (**c**) UV–vis absorption spectrum.

The schematic device structure, energy-level diagrams, and UV–vis absorption spectra are displayed in Figure 2, and the relevant photophysical and electrochemical parameters of the three small-molecule donors (TTiC12, TiC12, and TbC12) are shown in Table 1. The HOMO/LUMO energy levels of the three small-molecule donors were determined to be −5.29/−3.42 eV (TTiC12), −5.45/−3.26 eV (TiC12), and −5.45/−3.23 eV (TbC12). For reference, the corresponding values for PTB7-Th and PC_71_BM were −5.25/−3.55 eV and −5.96/−3.98 eV, respectively.

As shown in Table 1, the extinction coefficients of small-molecule donor TTiC12, TiC12, and TbC12 are 9.47 × 10^4^, 2.13 × 10^5^, and 1.34 × 10^5^ L mol^−1^ cm^−1^, respectively. The extinction coefficients of small-donor TiC12 and TbC12 with difluorobenzothiadiazole as the core were much higher than those of small-donor TTiC12 with pyrrole and pyrrole dione as the core. Regarding the absorption profiles, TTiC12 shows a maximum absorption peak (λ_max_) at 804 nm with a main absorption range of 550–800 nm, whereas TiC12 and TbC12 both display λ_max_ at 700 nm with absorption spanning 450–680 nm. For comparison, the polymer donor PTB7-Th, a narrow-bandgap material, exhibits λ_max_ at 780 nm and absorbs primarily in the 550–750 nm range, while the fullerene acceptor PC_71_BM shows relatively weak absorption above 550 nm. Consequently, the absorption of TTiC12 substantially overlaps with that of PTB7-Th, resulting in poor spectral complementarity. In contrast, the 450–680 nm absorption of TiC12 and TbC12 effectively compensates for the weaker absorption of PTB7-Th in this region, thereby enhancing the overall photon-harvesting capability of the blend film. This complementary absorption is expected to contribute positively to the short-circuit current density (*J*_sc_) of the corresponding ternary devices.

### 2.2. Photovoltaic Property

**Figure 3:**
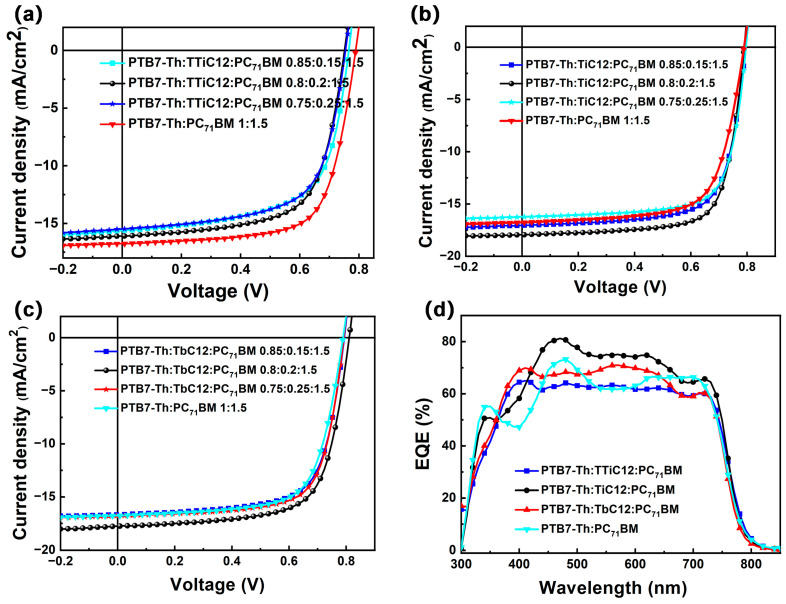
*J–V* curves of (**a**) PTB7-Th:TTiC12:PC_71_BM, (**b**) PTB7-Th:TiC12:PC_71_BM, (**c**) PTB7-Th:TbC12:PC_71_BM devices of different proportions and (**d**) EQE curves of the optimal binary and ternary OSCs.

To investigate the effect of the three small-molecule donors (TTiC12, TiC12, and TbC12) on device performance, the photoelectric properties of small-molecule donor TTiC12, TiC12 and TbC12 with different mass ratios were tested under a simulated light source with irradiation intensity of 100 mW cm^−2^ at AM 1.5 G (for more details see Supporting Information). The current density–voltage (*J–V*) curves of the optimized devices are presented in Figure 3, and the corresponding photovoltaic parameters are summarized in Tables S1–S3. Table 2 summarizes the key photovoltaic parameters of the optimized binary and ternary devices for direct comparison. The electron transport layer was Zracac, and the hole transport layer was PEDOT:PSS. The mass fraction of the small-molecule donor in the total donor (x = 0, 15, 20, 25 wt%) was systematically optimized.

As shown in Figure 3a, the ternary device with added small-molecule donor TTiC12 shows no improvement in *J*_sc_ compared to the binary PTB7-Th:PC_71_BM reference device. However, the optimal ratio of small-molecule donors TiC12 and TbC12 results in significant improvement in *J_sc_* compared to the binary reference device (Figure 3b,c). These results are consistent with the conclusion drawn in Figure 3d. The PTB7-Th:PC_71_BM component achieved an energy conversion efficiency of 9.16%, with a *V_oc_* of 0.789 V, a *J_sc_* of 16.75 mA cm^−2^ and a FF of 69.4%. The performance of the ternary device PTB7-Th:TTiC12:PC_71_BM with the addition of 20% small-molecule donor TTiC12 was significantly reduced, which is mainly attributable the severe spectral overlap between the absorption of TTiC12 and that of the polymer donor PTB7-Th. Compared to the reference binary device, it fails to effectively enhance absorption, and its lower extinction coefficient does not lead to an increase in *J_sc_*. Ultimately, these combined factors lead to a reduction in the PCE.

However, due to the excellent performance of the small-molecule donors TiC12 and TbC12, the performance of PTB7-Th:TiC12:PC_71_BM and PTB7-Th:TbC12:PC_71_BM containing 20% small-molecule donor TiC12 and TbC12 has been improved simultaneously. PCE increases from 9.16% to 10.42% and 9.99% for PTB7-Th:PC_71_BM components, respectively, and *J_sc_* increases from 16.75 mA cm^−2^ to 17.92 mA cm^−2^ and 17.77 mA cm^−2^, respectively. The main reason for the improvement of PCE of the device is that small-molecule donors TiC12 and TbC12 effectively enhance the absorption of the polymer PTB7-Th and the fullerene acceptor PC_71_BM. Coupled with their high extinction coefficients, this leads to a significant increase in *J_sc_*. Consequently, ternary devices incorporating the difluorobenzothiadiazole-core small-molecule donors TiC12 and TbC12 achieve a higher PCE compared to the reference binary device, demonstrating a 13.8% improvement in PCE.

To further understand the variation in *J_sc_* response intensity in different wavelength ranges, the external quantum efficiency (EQE) spectra of binary and ternary photovoltaic devices were tested, as shown in Figure 3d. For the TTiC12-based ternary device, the EQE response is enhanced in the 350–430 nm range compared with the binary PTB7-Th:PC_71_BM reference. This enhancement corresponds to the strong absorption of TTiC12 in this region, which effectively compensates for the relatively weak absorption of the fullerene acceptor PC_71_BM. However, in the wavelength ranges of 430–510 nm and 600–740 nm, the EQE response of the ternary blend is weaker than that of the binary counterpart. This reduction is attributed to the decreased relative content of the polymer donor PTB7-Th in the blend film upon the introduction of the third component, which diminishes the contribution of PTB7-Th to photocurrent generation in these regions. For the TiC12-based ternary device, a broad EQE enhancement is observed across the 360–680 nm range, which is consistent with the absorption profile of TiC12, indicating effective complementary photon harvesting by this small-molecule donor. In the case of the TbC12-based ternary device, the EQE response is enhanced in the 350–430 nm and 520–650 nm ranges, but weakened in the 430–520 nm and 650–750 nm ranges. This behavior can be rationalized by the relatively lower absorption intensity of TbC12 compared with PTB7-Th in both the shorter and longer wavelength regions, where the polymer donor remains the dominant light-absorbing component. Overall, the EQE results are in good agreement with the UV–vis absorption spectra (Figure 2c) and the *J–V* characteristics (Figure 3a–c), further corroborating the conclusions drawn from the photophysical and photovoltaic measurements.

### 2.3. Charge Dynamics

**Figure 4:**
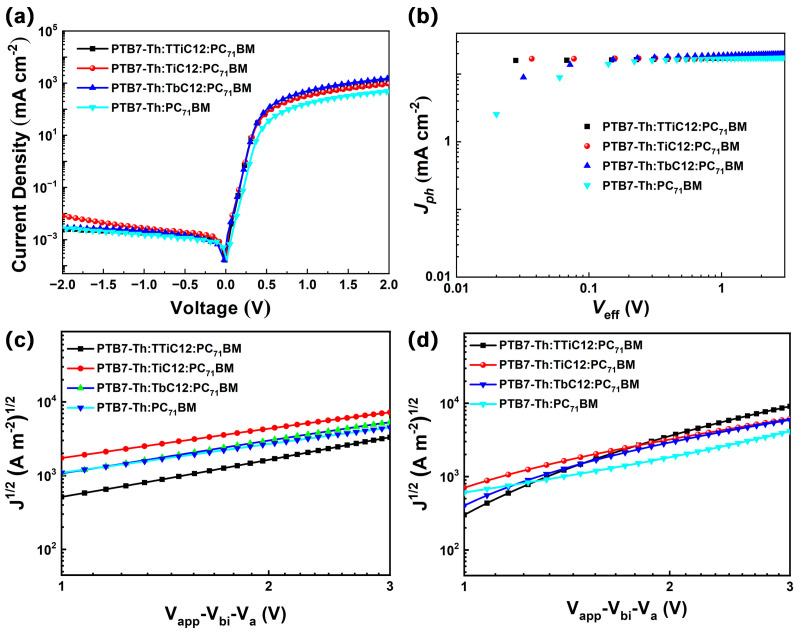
(**a**) Dark current; (**b**) the corresponding *J_ph_-V_eff_* curve; (**c**) the hole mobility of devices based on PTB7-Th:PC_71_BM system and (**d**) the corresponding electron mobility.

To gain insight into the charge recombination and leakage behavior, the dark-state current density–voltage (*J–V*) characteristics of the devices were measured, as shown in Figure 4a. Compared with the binary PTB7-Th:PC_71_BM reference, the ternary devices incorporating TiC12 and TbC12 exhibit significantly higher shunt resistance. This increased resistance is beneficial for suppressing leakage current and facilitating efficient carrier transport, thereby contributing to improved device performance. To further evaluate the exciton dissociation and charge collection efficiency, the photocurrent density (*J_ph_*) as a function of effective voltage (*V_eff_*) was measured, with the corresponding curves presented in Figure 4b. Here, *J_ph_* is defined as *J_L_-J_D_*, *J_L_* represents the short-circuit current density in bright state, *J_D_* represents the short-circuit current density in dark state, *V_eff_* is defined as *V_0_-V_appl_*, *V_0_* is the voltage value when *J_ph_* = 0, and *V_appl_* is the voltage value applied by the outside world during the test. As the voltage value applied by the outside world increases, the *J_ph_* will gradually approach the saturation current density (*J_sat_*), that is, the charge formed by all the excitons separated can be collected at the electrode under the action of the external electric field. In the short-circuit state, the *J_ph_/J_sat_* value can represent the exciton dissociation efficiency of the photovoltaic device. When a high bias voltage is applied, the carrier recombination inside the active layer will be inhibited, and the photogenerated carriers can be extracted smoothly, and the *J_ph_/J_sat_* value can reach 100% under ideal conditions without recombination [33]. For PTB7-Th:TTiC12:PC_71_BM, PTB7-Th:TiC12:PC_71_BM and PTB7-Th:TbC12:PC_71_BM three components compared with PTB7-Th:PC_71_BM two components, the *J_ph_/J_sat_* values are 95.99%, 97.60%, 96.37% and 95.43%, respectively. These results indicate that the ternary PTB7-Th:TiC12:PC_71_BM and PTB7-Th:TbC12:PC_71_BM devices exhibit significantly higher exciton dissociation efficiency compared to the binary PTB7-Th:PC_71_BM reference. *J_ph_/J_sat_* values at maximum power output indicate charge transfer and collection efficiency. According to the measured *J_ph_/J_sat_* values, it was found that PTB7-Th:TiC12:PC_71_BM ternary device has a higher exciton rate and exciton ionization rate, which is helpful to improve the *J_sc_* and FF of the device.

The carrier mobility of the device is further studied by using space-charge limited current method (SCLC). From the slope of the *J*_1/2_*-V* curve, we can obtain the desired mobility value. Figure 4c,d show the *J*_1/2_*-V* characteristic curves of hole devices and electronic devices of different materials, and the hole and electron mobility are shown in Table 3. As shown in Table 3, the hole mobilities of the PTB7-Th:PC_71_BM binary system and the PTB7-Th:TTiC12:PC_71_BM, PTB7-Th:TiC12:PC_71_BM, and PTB7-Th:TbC12:PC_71_BM ternary systems are 1.68 × 10^−4^, 1.21 × 10^−4^, 2.68 × 10^−4^, and 1.95 × 10^−4^ cm^2^ V^−1^ s^−1^, respectively. The corresponding electron mobilities are 1.60 × 10^−4^, 3.21 × 10^−4^, 2.18 × 10^−4^, and 2.30 × 10^−4^ cm^2^ V^−1^ s^−1^, respectively. The results indicate that the binary PTB7-Th:PC_71_BM system exhibits relatively low hole and electron mobilities, leading to severe charge recombination and a low FF. In contrast, the ternary devices, particularly the PTB7-Th:TiC12:PC_71_BM system, achieve simultaneously high hole and electron mobilities. When the hole and electron mobilities are balanced (μ_h_ ≈ μ_e_), space charge accumulation is minimized and recombination losses are effectively suppressed. These findings demonstrate that the introduction of thieno-isobenzopyrane small-molecule donors TiC12 and TbC12, featuring difluorobenzothiadiazole as the core, into the PTB7-Th:PC_71_BM system, can significantly improve carrier mobility, enhance charge transport and collection, and ultimately boost device performance. It is noteworthy that although the TTiC12-based ternary blend exhibits the highest electron mobility, it yields the lowest PCE. This discrepancy suggests that while the TTiC12 molecules facilitate efficient electron transport due to their strong crystallization tendency and favorable local π-π stacking, the resulting phase separation limits the donor–acceptor interfacial area. This morphological defect hinders exciton dissociation efficiency, thereby reducing the *J_sc_* and FF, which outweighs the benefit of high carrier mobility.

### 2.4. Active Layer Morphology

**Figure 5:**
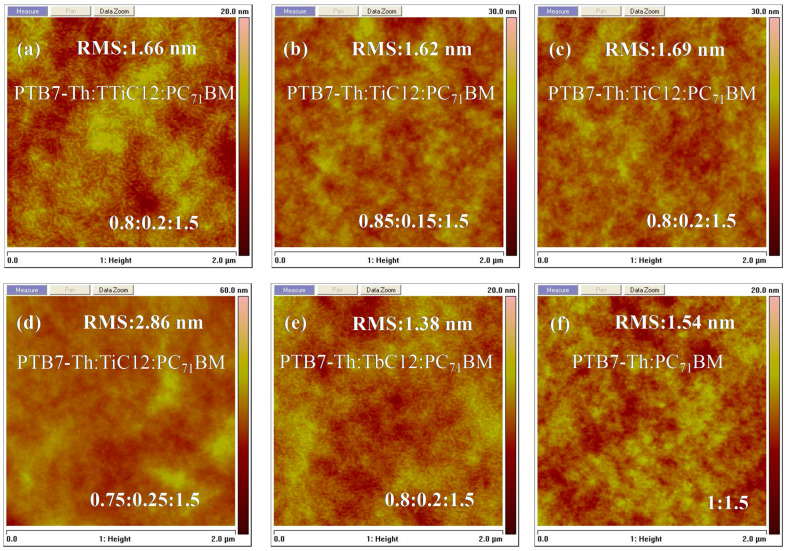
AFM morphology of (**a**) PTB7-Th:TTiC12:PC_71_BM, (**b**–**d**) PTB7-Th:TiC12:PC_71_BM, (**e**) PTB7-Th:TbC12:PC_71_BM and (**f**) PTB7-Th:PC_71_BM blend films with different proportions.

To investigate the influence of the three small-molecule donors on the film microstructure, atomic force microscopy (AFM) was performed on the blend films, with the resulting topographic images presented in Figure 5. Among them, the roughness of the PTB7-Th:TTiC12:PC_71_BM blended film is 1.66 nm (Figure 5a), and the roughness of the PTB7-Th:PC_71_BM blended film is 1.54 nm (Figure 5f), and the roughness of the two blended films is not much different. However, the PTB7-Th:TTiC12:PC_71_BM blend exhibits a higher degree of aggregation compared to the PTB7-Th:PC_71_BM binary blend, which is detrimental to charge collection, transport, and exciton dissociation. The roughness of the PTB7-Th:TbC12:PC_71_BM blend film is 1.38 nm (Figure 5e), which is smaller than that of the PTB7-Th:PC_71_BM blend film, and its surface topography is smoother and has fewer surface defects. However, excessive film-forming ability is detrimental to the formation of an appropriate phase separation. The PTB7-Th:TiC12:PC_71_BM blend film exhibits a roughness of 1.69 nm (Figure 5c) and a more uniform morphology compared to the PTB7-Th:PC_71_BM blend film. This moderate roughness facilitates an increase in the donor–acceptor interfacial area, leads to a more uniform phase distribution, and effectively prevents the aggregation of PC_71_BM [34]. Consequently, an interpenetrating network structure is formed, which is highly conducive to improving device performance.

To further optimize the TiC12 loading, AFM measurements were conducted on PTB7-Th:TiC12:PC_71_BM blend films with different TiC12 mass fractions (15, 20, and 25 wt%) (Figure 5b–d). It was found that the morphology of the films was less affected when 15% and 20% small-molecule donor TiC12 were added. All of them can form an interpenetrating network structure, which is conducive to charge collection and exciton separation, and increase short-circuit current. When 25% small-molecule donor TiC12 is added, its morphology changes greatly, and the roughness increases to 2.86 nm while the aggregation degree becomes obvious, which is not conducive to the improvement of device performance. These results indicate that regulating the appropriate proportion of small-molecule donor TiC12 is an effective way to optimize the morphology.

To further investigate the microscopic morphology of the active layer after aging, we conducted additional AFM measurements on the blend films after thermal aging at 100 °C in a nitrogen-filled glovebox and the results are shown in Figure S1. For the binary control device (without the third component): The AFM images reveal significant phase separation and the formation of large fullerene aggregates after aging, leading to a substantial increase in root-mean-square roughness (RMS) from 1.54 nm to 1.84 nm. This indicates severe morphological degradation. For the ternary device (TiC12): Remarkably, the film maintains a smooth and uniform interpenetrating network structure even after the same aging process. The RMS value only slightly increased from 1.69 nm to 1.71 nm, and no obvious large-scale aggregation was observed. For the ternary devices (TTiC12 and TbC12), their RMS values only slightly increased from 1.66 and 1.38 to 1.68 and 1.42, respectively. These microscopic observations provide direct visual evidence that the incorporation of the third component effectively suppresses the diffusion and aggregation of fullerene molecules, thereby locking in the optimal morphology and enhancing the long-term stability of the devices.

### 2.5. Device Stability

**Figure 6:**
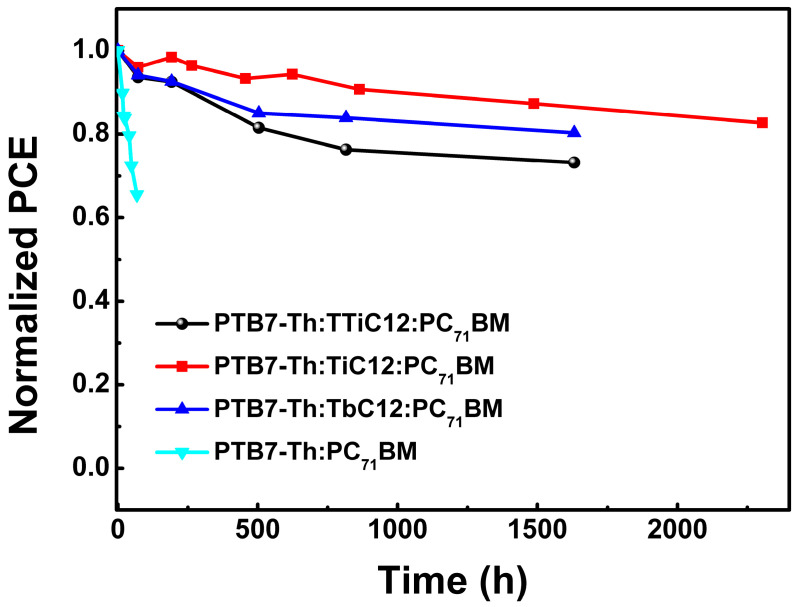
Time stability of devices in PTB7-Th:PC_71_BM system under nitrogen environment.

The long-term stability of the binary and ternary devices was evaluated by monitoring the PCE decay over time during storage in a N_2_-filled glovebox, as shown in Figure 6. Fullerene-based OSCs are known to suffer from morphological degradation over time due to the intrinsic aggregation tendency of fullerene acceptors, which leads to performance deterioration [35]. As shown in Figure 6, the binary PTB7-Th:PC_71_BM control device degrades rapidly, retaining only 65.5% of its initial PCE after 68 h of storage. In contrast, the ternary devices exhibit substantially enhanced stability. The TTiC12-based ternary device maintains 73.2% of its initial efficiency after 1632 h. The TbC12-based device shows even better stability, retaining 80.3% of its initial PCE over the same 1632 h period.

Remarkably, the TiC12-based ternary device achieves the best overall stability, preserving 90.7% of its initial efficiency after 864 h and still maintaining 82.7% after 2304 h. The pronounced stability improvement observed in the ternary systems is primarily attributed to the morphological stabilization imparted by the small-molecule donors. The pristine PTB7-Th:PC_71_BM blend suffers from severe aggregation of the spherical fullerene acceptor PC_71_BM, which disrupts the original film morphology and causes rapid device degradation. However, the introduction of TTiC12, TiC12, or TbC12 effectively suppresses such aggregation. Owing to their intrinsic molecular crystallinity, these small-molecule donors enhance the overall crystallinity of the blend film and optimize the phase separation scale upon incorporation into the binary host. Consequently, a more ordered molecular arrangement and a stable interpenetrating network with well-defined phase distribution are established in the active layer, which significantly retards the morphological degradation and extends the device lifetime.

## 3. Materials and Methods

PTB7-Th and PC_71_BM were purchased from J&K Scientific Co., Ltd., Beijing, China. All general analytical-grade reagents, such as chlorobenzene (CB) and 1,8-diiodooctane (DIO), were purchased from Sigma-Aldrich (Shanghai, China) and used without further purification. Poly(3,4-ethylenedioxythiophene) polystyrene sulfonate (PEDOT:PSS) (Clevios PVP Al 4083) was obtained from H.C. Starck, Shanghai, China. Molybdenum oxide (MoO_3_), Zracac and aluminum (Al) were purchased from Alfa Aesar Co., Ltd., Shanghai, China. All materials were used without further purification. According to our previous study [19] and synthetic routes [21], the three small-molecule donors TTiC12, TiC12, and TbC12 were prepared through sequential bromination, Grignard addition, Stille cross-coupling, and terminal Knoevenagel condensation. The full synthetic procedures can be found in our prior report [21]. More Materials and Methods details in Supplementary Materials. The detailed device fabrication is also presented in Supplementary Materials.

## 4. Conclusions

In summary, we have systematically investigated the influence of three structurally related small-molecule donors, TiC12, TbC12, and TTiC12, as third components on the photovoltaic performance and stability of the PTB7-Th:PC_71_BM host system. Among these, the ternary device incorporating TiC12 delivered the best performance, achieving a PCE of 10.42%, which represents a notable improvement over the 9.16% of the binary PTB7-Th:PC_71_BM reference. The TbC12-based ternary system also exhibited enhanced performance, with a PCE of 9.99%, attributable to its structural similarity to TiC12. In contrast, the TTiC12-containing ternary device showed no increase in short-circuit current density, as its absorption profile largely overlaps with that of the binary host. Moreover, this system suffered from a significant energy-loss penalty, resulting in a reduced open-circuit voltage from 0.789 V to 0.751 V, and consequently, a decreased PCE of 7.99%. More importantly, the ternary devices exhibited substantially enhanced long-term stability. The TiC12-based device retained 82.7% of its initial PCE after 2304 h of storage in a N_2_-filled glovebox, while the TbC12-based device maintained 80.3% of its initial efficiency after 1632 h under identical conditions. The TTiC12-based device, by comparison, preserved 73.2% of its initial PCE over the same 1632 h period. Strikingly, the binary PTB7-Th:PC_71_BM control device degraded rapidly, retaining only 65.5% of its initial efficiency after just 68 h. Further morphological characterization revealed that the incorporation of TiC12 and TbC12 effectively modulates the crystallinity of the active layer, promotes favorable intermolecular stacking, and optimizes the nanoscale phase separation of the PTB7-Th:PC_71_BM blend. More critically, the binary host film is prone to severe aggregation over time; the introduction of these small-molecule donors, particularly those bearing the difluorobenzothiadiazole (DFBT) core, effectively suppresses such aggregation and stabilizes the desired morphology, thereby accounting for the concurrent enhancement in both efficiency and stability. Our findings demonstrate that rational design of small-molecule donors as ternary components represents a viable and powerful strategy for simultaneously boosting the PCE and operational lifetime of organic solar cells, bringing them a step closer to practical applications.

## Figures and Tables

**Table 1:** Photophysical and electrochemical properties of TTiC12, TiC12 and TbC12.

Donors	*λ_max_* ^a^ [nm]	*ε_max_* ^a^ [L mol^−1^ cm^−1^]	*λ_max_* ^b^ [nm]	*λ_onset_* ^b^ [nm]	*E_g_^opt^* [eV]	*E_HOMO_* [eV]	*E_LUMO_* [eV]	*E_g_^CV^* [eV]
TTiC12	632	9.47 × 10^4^	752	804	1.54	−5.29	−3.42	1.87
TiC12	578	2.13 × 10^5^	580	700	1.77	−5.45	−3.26	2.19
TbC12	575	1.34 × 10^5^	585	700	1.77	−5.45	−3.23	2.23

^a^ Absorption in trichloromethane solution; ^b^ absorption in the film state.

**Table 2:** The photovoltaic parameters of optimized binary and ternary OSCs.

Active Layer Components	D:A Ratio	*V*_oc_ [V]	*J*_sc_ [mA cm^−2^]	FF [%]	PCE [%]
PTB7-Th:PC_71_BM	1:1.5	0.789	16.75	69.4	9.16
PTB7-Th:TTiC12:PC_71_BM	0.8:0.2:1.5	0.751	16.08	66.1	7.99
PTB7-Th:TiC12:PC_71_BM	0.8:0.2:1.5	0.790	17.92	73.6	10.42
PTB7-Th:TbC12:PC_71_BM	0.8:0.2:1.5	0.790	17.77	69.4	9.99

**Table 3:** Hole and electron mobility of devices based on PTB7-Th:PC_71_BM system.

Active Layer	Hole Mobility (cm^2^ V^−1^ s^−1^)	Electron Mobility (cm^2^ V^−1^ s^−1^)
PTB7-Th:TTiC12:PC_71_BM	1.21 × 10^−4^	3.21 × 10^−4^
PTB7-Th:TiC12:PC_71_BM	2.68 × 10^−4^	2.18 × 10^−4^
PTB7-Th:TbC12:PC_71_BM	1.95 × 10^−4^	2.30 × 10^−4^
PTB7-Th:PC_71_BM	1.68 × 10^−4^	1.60 × 10^−4^

## Data Availability

The original contributions presented in this study are included in the article. Further inquiries can be directed to the corresponding authors.

## Supplementary Materials

The following supporting information can be downloaded at: https://www.mdpi.com/article/10.3390/molecules31142552/s1, General Materials; Synthesis of TTiC12, TiC12 and TbC12; General Measurements; OSC Device Fabrication and Measurement; Table S1. Parameters of PTB7-Th:TTiC12:PC_71_BM devices of different proportions; Table S2. Performance parameters of PTB7-Th:TiC12:PC_71_BM devices of different proportions; Table S3. Performance parameters of PTB7-Th:TbC12:PC_71_BM devices of different proportions; Figure S1. (a) AFM height images of PTB7-Th:TTiC12:PC_71_BM blend films under thermal annealing at 100 °C. (b) AFM height images of PTB7-Th:TiC12:PC_71_BM blend films under thermal annealing at 100 °C. (c) AFM height images of PTB7-Th:TbC12:PC_71_BM blend films under thermal annealing at 100 °C. (d) AFM height images of PTB7-Th:PC_71_BM blend films under thermal annealing at 100 °C; Refs. [19,21] are cited in the Supplementary Materials.
