# Supplementary material for: Simultaneously Enhancing Efficiency and Stability of Ternary Organic Solar Cells via a Benzothiadiazole-Thieno[3,2-c]isochromene-Based Small-Molecule Donor in the PTB7-Th:PC71BM System
Source: Molecules. 2026 Jul 22;31(14):2552. doi: 10.3390/molecules31142552 (PMC13415099; doi:10.3390/molecules31142552)
Supplement: Supplementary file 1 [file molecules-31-02552-s001.zip › molecules-4457608-supplementary.pdf]

Supporting Information

Simultaneously Enhancing Efficiency and Stability of Ternary Organic Solar Cells via a Benzothiadiazole-Thieno[3,2-c]isochromene-Based Small-Molecule Donor in the PTB7-Th:$PC_{71}BM$ System

Wei Tang, Wenjie Zeng, Junjie Liu, Junhui Zhou and Xiaobing Lan *

Hunan Provincial Key Laboratory of Xiangnan Rare-Precious Metals Compounds Research and Application, School of Chemistry and Environmental Science, Xiangnan University, Chenzhou 423000, China

* Correspondence: xblan@xnu.edu.cn

Contents

1 General Materials....................2
2 Synthesis of TTiC12, TiC12 and TbC12....................2
3 General Measurements....................3
4 OSC Device Fabrication and Measurement....................4
5 Additional OSC Device Performance Data....................5
6 Atomic Force Microscopy (AFM)....................6
7 References....................6

1. General Materials

Chlorobenzene (CB) and 1, 8-diiodooctane (DIO) were purchased from Aldrich and used without further purification. PTB7-Th and $PC_{71}BM$ were purchased from J&K Scientific Co., Ltd. Poly(3,4-ethylenedioxythiophene) polystyrene sulfonate (PEDOT:PSS) (Clevios PVP Al 4083) was obtained from H.C. Starck, Germany. Molybdenum oxide (MoO_3), Zracac and aluminum (Al) were purchased from Alfa Aesar Co., Ltd. All materials were used without further purification. TTiC12, TiC12 and TbC12 was synthesized by our research group.

Solution route: Preparation of the active layer: The small molecule donor materials TTiC12, TiC12 and TbC12 were added to the polymer donor PTB7-Th and the fullerene receptor $PC_{71}BM$ respectively, and dissolved in chlorobenzene with A mass ratio of D/A of 1-x:x:1.5 ($x<1$), with a total solubility of 25 mg/mL. Place on a 60 °C heating panel and stir overnight. After stirring, add 3% 1, 8-diiodooctane. PTB7-Th:TTiC12:$PC_{71}BM$, PTB7-Th:TiC12:$PC_{71}BM$ and PTB7-Th:TbC12:$PC_{71}BM$ active layer solution are configured. Preparation of electron transport layer zinc oxide (ZnO):110 mg zinc acetate dihydrate particles and 31 mg ethanolamine were dissolved in 1 mL 2-methoxyethanol solution and stirred at room temperature for 24 h. The prepared solution was dropped on the surface of ITO glass and spun on it for 30 s at a rotational speed of 3000 rpm/s, and then the spun ZnO substrate was annealed on a heated panel at 150 °C for 30 min. Preparation of electron transport layer Zirconium acetyl acetone (Zracac):Take 4 mg zirconium acetyl acetone solid powder into a glass bottle, add 4 mL anhydrous ethanol, stir overnight at room temperature.

2. Synthesis of TTiC12, TiC12 and TbC12

According to our previous study[1] and the synthetic routes[2], the small donor TTiC12, TiC12 and TbC12 were prepared via Stille coupling, bromination, Grignard reaction, and Knoevenagel condensation. Details as follows:

TTiC12: In a 50 mL two-necked round-bottomed flask, dissolve compound 8[2] (120 mg, 0.07 mmol) and inden-2-one (102 mg, 0.7 mmol) in an appropriate amount

of anhydrous chloroform. Add 1 drop of triethylamine, mix magnetically, and react under nitrogen protection for 24 hours. After adding ethanol to cause sedimentation, separate and purify the product using column chromatography with a mixed solvent of petroleum ether/dichloromethane (v/v, 1:1), dichloromethane, and trichloromethane as eluents. This yields a black solid, 110 mg, with a yield of 82.9%. ^{1}H NMR (400 MHz, $CDCl_3$) 8 9.04 (d, *J*= 4.1 Hz, 2H), 8.00 - 7.93 (m, 4H), 7.84 (s, 2H), 7.82 -7.75 (m, 4H), 7.64 (s, 2H), 7.60 (d, *J*= 8.1 Hz, 2H), 7.53 -7.48 (m, 4H), 7.33 (s, 2H), 4.17 - 4.07 (m, 4H), 2.06 - 1.90 (m, 10H), 1.46 - 1.17 (m, 96H),0.96 (t, *J*=7.4 Hz, 6H), 0.89 (t, *J*= 7.0 Hz, 6H), 0.85 (t, *J*= 6.8 Hz, 12H).

TiC12: To a solution of compound 8[2] (100 mg， 0.07 mmol), 1,3-indanedione (112.5 mg, 0.77 mmol) and dry $CHCl_3$ (25 mL) in a two-neck round-bottom flask, a drop of triethylamine was added in a 50 mL flask. The mixture was stirred for 24 h at room temperature under nitrogen atmosphere and concentrated by distillation. The concentrated solution was dropped into ethanol to form precipitation. The precipitation was collected and purified by silica column using chloroform as an eluent. And TiC12 was obtained as black solid in a yield of 89.3%. It further purified by re-precipitation by adding its concentration $CHCl_3$ solution into ethanol. ^{1}H NMR (400 MHz, $CDCl_3$) δ 8.26 (d, *J* = 3.8 Hz, 2H), 7.93 (d, *J* = 7.6 Hz, 2H), 7.90 (d, *J* = 6.2 Hz, 2H), 7.73 (s, 6H), 7.61 (s, 2H), 7.53 (d, *J* = 7.9 Hz, 2H), 7.41 (d, *J* = 3.9 Hz, 2H), 7.38 (d, *J* = 7.9 Hz, 2H), 7.35 (s, 2H), 2.10 – 1.92 (m, 8H), 1.50 – 1.21 (m, 80H), 0.84 (t, *J* = 6.7 Hz, 12H).

TbC12: To a solution of compound 8[2] (100 mg ， 0.07 mmol) and 2-(3-ethyl2-oxo-4-thiazolidinylidene) propanedinitrile (148.8 mg, 0.77 mmol) in dry $CHCl_3$ (25 mL), a drop of triethylamine was added in a round-bottom flask. The mixture was stirred for 24 h at room temperature under nitrogen and concentrated by distillation. The concentrated solution was dropped into ethanol to get precipitation. The precipitation was collected and purified by silica column using chloroform as an eluent. And TbC12 was obtained as black solid in a yield of 89.4%. It further purified by re-precipitation by adding its concentration $CHCl_3$ solution into ethanol. ^{1}H NMR (400 MHz, $CDCl_3$) 8 9.02 (d, *J*=4.1 Hz, 2H), 7.96 (s, 2H),7.60 (dd, *J*= 8.0, 1.5 Hz,

2H), 7.50 (d, *J*= 4.2 Hz, 2H), 7.38 (d, *J*= 8.1 Hz, 2H), 7.32(s, 2H), 7.04 (s, 2H), 4.33 (q, *J* = 7.0 Hz, 4H), 4.16 - 4.06 (m, 4H), 2.05 - 1.90 (m,10H), 1.42 (t, *J*=7.2 Hz, 12H), 1.37 - 1.17 (m, 90H), 0.95 (t, *J*=7.4 Hz, 6H), 0.87 (dt, *J*= 10.1,6.9 Hz, 18H).

3. General Measurements

UV-vis absorption spectra were obtained using a Shimadzu UV-1800 PC spectrometer. The electrochemical cyclic voltammetry (CV) was measured with a CHI630E electrochemical workstation with the working electrode dipped in an acetonitrile solution of 0.1 M tetrabutyl- ammonium hexafluorophosphate (Bu_4NPF_6) under nitrogen at room temperature.

4. OSC Device Fabrication and Measurement

The organic solar cells were fabricated with a conventional configuration of ITO /PEDOT: PSS (30 nm) /active layer (100 nm) /Zracac (10 nm) /Al (100 nm). The cell area is 0.06 cm^2. The used indium tin oxide (ITO)-glass substrates were sequentially washed by Decon90 dilution solution, deionized water, acetone and isopropyl alcohol using ultrasonic process for each 20 min, respectively, then were treated by UV-ozone for 15 min. PEDOT:PSS was spin-coated at 4800 rpm for 30 s on the cleaned ITO substrate and annealed at 150 °C for 15 min in air. The substrates were transferred into an N_2-filled glove box for preparing the photoactive layer. The PTB7-Th:$PC_{71}BM$ blend solutions were prepared using chlorobenzene solvent. The PTB7-Th concentration in the blend solution was 10 mg mL^{-1} (donor: acceptor = 1:1.5 by weight). The additive ratio of DIO was 3% by volume. The blend solutions were stirred for 12 h before use. In the ternary blends, the doping ratios of TTiC12, TiC12 and TbC12 in the PTB7-Th:$PC_{71}BM$ blend were 15%, 20% and 25% by weight. The binary and ternary active layers were spin-coated on PEDOT: PSS with the resulting thickness of 130 nm and stayed in a vacuum for half an hour. Finally, the Zracac (10 nm) and Al (100 nm) electrodes were thermally deposited with a shadow mask of 0.06 cm^2 at the pressure of 10^{-4} Pa. The current density-voltage (*J-V*) characteristics were performed using a programmable Keithley 2400 source measurement unit under

simulated solar light (AM 1.5 G) (DM40S3, SAN-EI ELECTRIC, Japan). The light intensity was determined by the standardized mono-silicon cell (Oriel PN 91150V, Newport, USA). The external quantum efficiency (EQE) spectra were obtained by a photo-modulation spectroscopic setup (Newport monochromator).

Space charge limited current (SCLC) method was applied to determine the electron and hole mobilities in the electron-only device with a structure of ITO/ZnO/active layer/Zracac/Al and hole-only device with a structure of ITO/PEDOT:PSS/active layer/MoO_3/Al, respectively. The mobility was determined by the following equation:

$$J = \frac{9}{8}\mu\varepsilon\varepsilon_0\frac{V^2}{L^3} \quad (1)$$

Where ε is the dielectric permittivity of the polymer (generally taken to be about 3), ε_0 is the dielectric permittivity of free space, L is the film thickness, and V is the voltage, which is defined as $V = V_{appl} - V_{bi}$, where V_{appl} is the applied voltage, V_{bi} is the built-in voltage, which is related to the difference in the work function of the electrodes.

5. Additional OSC Device Performance Data

Table S1. Parameters of PTB7-Th:TTiC12:$PC_{71}BM$ devices of different proportions.

PTB7-Th:TTiC12:$PC_{71}BM$	V_{oc} [V]	J_{sc} [mA cm^{-2}]	FF [%]	PCE [%]
1:0:1.5	0.789	16.75	69.4	9.16
0.85:0.15:1.5	0.766	15.61	63.8	7.62
0.8:0.2:1.5	0.751	16.08	66.1	7.99
0.75:0.25:1.5	0.755	15.47	65.0	7.59

Table S2. Performance parameters of PTB7-Th:TiC12:$PC_{71}BM$ devices of different proportions.

PTB7-Th:TiC12:$PC_{71}BM$	V_{oc} [V]	J_{sc} [mA cm^{-2}]	FF [%]	PCE [%]
1:0:1.5	0.789	16.75	69.4	9.16
0.85:0.15:1.5	0.795	17.04	71.4	9.68
0.8:0.2:1.5	0.790	17.92	73.6	10.42

0.75:0.25:1.5	0.796	16.23	73.1	9.45

Table S3. Performance parameters of PTB7-Th:TbC12:$PC_{71}BM$ devices of different proportions.

PTB7-Th:TbC12:$PC_{71}BM$	V_{oc} [V]	J_{sc} [mA cm^{-2}]	FF [%]	PCE [%]
1:0:1.5	0.789	16.75	69.4	9.16
0.85:0.15:1.5	0.793	16.65	70.0	9.24
0.8:0.2:1.5	0.810	17.77	69.4	9.99
0.75:0.25:1.5	0.792	16.78	71.4	9.48

6. Atomic Force Microscopy (AFM)

AFM measurements were performed in ambient on a SPID Bruker ICON using a TESPA probe. All the AFM images were flattened and exported from the NanoScope Analysis 1.9 software, the root-mean-square roughness (Rq) values of height images were obtained from the whole scan area (2 μm × 2 μm).

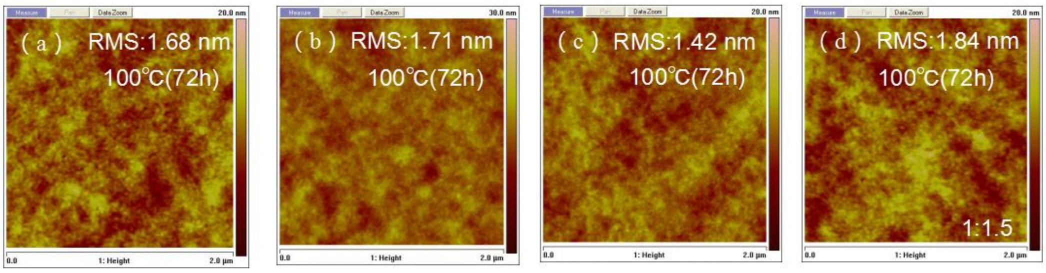

Figure S1. (a) AFM height images of PTB7-Th:TTiC12:$PC_{71}BM$ blend films under thermal annealing at 100 °C. (b) AFM height images of PTB7-Th:TiC12:$PC_{71}BM$ blend films under thermal annealing at 100 °C. (c) AFM height images of PTB7-Th:TbC12:$PC_{71}BM$ blend films under thermal annealing at 100 °C. (d) AFM height images of PTB7-Th:$PC_{71}BM$ blend films under thermal annealing at 100 °C.

7. References

[1] Tang, W.; Peng, W.; Zhu, M.; Jiang, H.; Wang, W.; Xia, H.; Yang, R.; Inganäs, O.; Tan, H.; Bian, Q.; Wang, E.; Zhu, W. 17.25% high efficiency ternary solar cells with increased open-circuit voltage using a high HOMO level small molecule guest donor

in a PM6:Y6 blend. *J. Mater. Chem. A* **2021,** *9,* 20493-20501.

[2] Wang, W.; Zhang, G.; Guo, J.; Gu, Z.; Hao, R.; Lin, Z.; Qian, Y.; Zhu, M.; Xia, H.; Peng, W.; Liu, X.; Peng, Q.; Zhu, W., Medium-Bandgap (Acceptor′–Donor)2Acceptor-Type Small-Molecule Donors Based on an Asymmetric Thieno[3,2-c]isochromene Building Block for Organic Solar Cells with High Efficiency and Voltage. *ACS Appl. Energy Mate.* **2019**, 2, 4730-4736.
